# High-Temperature Water Electrolysis Properties of Membrane Electrode Assemblies with Nafion and Crosslinked Sulfonated Polyphenylsulfone Membranes by Using a Decal Method

**DOI:** 10.3390/membranes14080173

**Published:** 2024-08-08

**Authors:** Je-Deok Kim

**Affiliations:** Environmental Circulation Composite Materials Group, Functional Materials Field, Research Center for Electronic and Optical Materials, National Institute for Materials Science (NIMS), 1-1 Namiki, Tsukuba 305-0044, Japan; kim.jedeok@nims.go.jp; Tel.: +81-29-860-4764; Fax: +81-29-860-4984

**Keywords:** high temperature, polymer electrolyte water electrolysis, nafion membrane, SPPSU polymer, CSPPSU membrane, decal method, CCM

## Abstract

To improve the stability of high-temperature water electrolysis, I prepared membrane electrode assemblies (MEAs) using a decal method and investigated their water electrolysis properties. Nafion 115 and crosslinked sulfonated polyphenylsulfone (CSPPSU) membranes were used. IrO_2_ was used as the oxygen evolution reaction (OER) catalyst, and Pt/C was used as the hydrogen evolution reaction (HER) catalyst. The conductivity of the CSPPSU membrane at 80 °C and 90% RH (relative humidity) is about four times lower than that of the Nafion 115 membrane. Single-cell water electrolysis was performed while measuring the current density and performing electrochemical impedance spectroscopy (EIS) at cell temperatures from 80 to 150 °C and the stability of the current density over time at 120 °C and 1.7 V. The current density of water electrolysis using Nafion 115 and CSPPSU membranes at 150 °C and 2 V was 1.2 A/cm^2^ for both. The current density of the water electrolysis using the CSPPSU membrane at 120 °C and 1.7 V was stable for 40 h. The decal method improved the contact between the CSPPSU membrane and the catalyst electrode, and a stable current density was obtained.

## 1. Introduction

Abnormal phenomena caused by global climate changes are leading to an economic society that can provide a stable energy supply while reducing CO_2_ emissions on a global scale. It is hoped that a sustainable energy society will be built through storage and conversion technology using naturally derived energy, such as sunlight, wind, and biomass. Hydrogen is suitable as an energy storage and transport carrier, and it can be produced from renewable energy, biomass, water electrolysis, etc., has a high energy weight density, and is suitable for long-term energy storage systems. The most significant advantage of hydrogen is that it emits zero CO_2_ when burned and can significantly contribute to achieving carbon neutrality. The New Energy and Industrial Technology Development Organization (NEDO) predicts that the hydrogen market will reach 160 trillion yen by 2050 [1]. In recent years, research on the production of green hydrogen by water electrolysis [2], photolysis [3], biomass conversion [4], and thermochemical methods [5] has become active. Among these green hydrogen production methods, water electrolysis systems could absorb the surplus electricity and output fluctuations from renewable energy sources, enable the further expansion of renewable energy use, and contribute to the realization of carbon neutrality. Water electrolysis includes polymer electrolyte membrane water electrolysis (PEMWE) [2], anion exchange membrane water electrolysis (AEMWE) [6], alkaline solution water electrolysis (AWE) [7], and solid oxide water electrolysis [8], which aim to reduce costs, increase durability, and improve efficiency. Polymer ion exchange electrolyte membranes are used in water electrolysis (H^+^, OH^−^) [2,6], fuel cells (H^+^, OH^−^) [9,10], batteries [11,12], redox flow batteries (RFBs) (H^+^, OH^−^) [13,14,15], and solar cells [16] and are required to have improved conductivity, improved mechanical and chemical properties, and lower cost.

PEMWE uses a proton exchange polymer electrolyte membrane as the electrolyte membrane, platinum, which is a hydrogen evolution reaction (HER) catalyst, as the cathode catalyst, and iridium (iridium oxide) and ruthenium (ruthenium oxide), which are oxygen evolution reaction (OER) catalysts, as the anode catalyst electrode [2]. Fluorine-based polymers, non-fluorine-based polymers, and composite polymers are being studied as proton-exchange polymer electrolyte membranes, and efforts are being made to find electrolyte membranes that can withstand the high temperatures of electrolytic systems [17]. PEMWE’s high-temperature operation (100–200 °C) should significantly improve the catalytic activity and the electrolyte membrane conductivity and reduce membrane–electrode interfacial resistance due to kinetic and thermodynamic advantages. Therefore, the overvoltage of the entire water electrolysis cell should be significantly reduced, improving the system’s efficiency [18,19,20,21]. However, an electrolyte membrane that can withstand high-temperature operation has not yet been put into practical use. Therefore, there are many unknowns, such as membrane properties and catalyst properties during high-temperature operation.

It has been reported that water electrolysis performance varies depending on the MEA manufacturing method [22,23,24,25,26]. There are two manufacturing methods for PEMWE-MEAs. One is the CCM method. In the CCM method, the catalyst layers are either directly coated on the membrane surface or transferred to the membrane surface by a decal process [22,23,24,25,26]. An alternative to the CCM method is the PTE method [18,19,24,25]. The PTE method faces the issue of high resistance when assembling the membrane and electrode [18,19]. In the CCM method, the interfacial resistance can be reduced by directly coating the surface of the membrane or by transferring it using the decal method, which allows for good contact between the membrane and the catalyst. Therefore, it has been reported that using the CCM method leads to a better water electrolysis performance than the PTE method [24].

In our previous paper [18,19], we used an IrO_2_ catalyst (7.5 mg/cm^2^) as a porous transport electrode (PTE) attached to a porous Ti electrode by electroplating the OER electrode. In addition, the HER electrode used a Pt/C (pt: 0.3 mg/cm^2^) catalyst coated with a gas diffusion layer (GDL). Nafion 115 and CSPPSU membranes were used as electrolyte membranes. In a high-temperature water electrolysis evaluation of a single cell, the current density at constant voltage decreased rapidly, and stability over time was an issue. The main reason is the connection between the membrane and the electrode. The PTE was hard and did not stick to the CSPPSU electrolyte membrane when the hot-press method was used. Therefore, it is thought that the stability of the current density over time at a constant voltage could not be obtained.

In this paper, a decal method, which is a catalyst-coated membrane (CCM) method, was used instead of the IrO_2_/Ti PTE. An IrO_2_ catalyst or Pt/C slurry was prepared on a Teflon sheet. These were transferred to a Nafion 115 membrane or a CSPPSU electrolyte membrane using a hot press to prepare membrane electrode assemblies (MEAs). The water electrolysis properties, such as current density and stability over time, of the MEAs using a decal method improved.

## 2. Experimental Section

The electrolyte membranes used were Nafion 115 (ChemoursTM, Wilmington, DE, USA) and CSPPSU. PPSU (Solvay Radel R-5000 NT) (Mw = 50,000) was provided by Solvay Specialty Polymers Japan K.K. (glass transition temperature (T_g_) = 220 °C). Sodium hydroxide (NaOH), Sulfuric acid (H_2_SO_4_), and sodium chloride (NaCl) were purchased from Nacalai Tesque, Inc, Japan. The treatment method for the Nafion 115 membrane has been described in a previous paper [18]. In addition, the synthesis of SPPSU membranes and the preparation of CSPPSU membranes were performed using the same method as used in previous papers [19,27]. Activation treatment was performed using the same method. Briefly, the crosslinking step was performed using heat treatment at 120 (24 h), 160 (24 h), and 180 °C (24 h) in this order. After cross-linking, the membranes were treated with 0.5 M NaOH (85–87 °C, 7 h), boiling water (2 h), 1 M H_2_SO_4_ (80 °C, 2 h), and boiling water (2 h) and finally dried at room temperature.

The ion exchange capacity (IEC) was defined as the milli-equivalent value of sulfonic acid groups per gram of dry sample. A portion of the membrane was immersed in 20 mL of 2 M NaCl solution and equilibrated for over 24 h to replace protons with sodium ions. This solution was then titrated with 0.01 M NaOH solution. The IEC value was calculated using the following formula:

IEC (meq/g) = CV/W_dry_

where C (mmol/L) is the concentration of the standardized NaOH aqueous solution used in the titration (0.01 mol/L), V (L) is the volume of the standardized NaOH aqueous solution used in the titration, and W_dry_ (g) is the mass of the dry membrane.

The degree of sulfonation (D.S.) of the SPPSU polymer was calculated using the following formula:

D.S. (sulfonic acid group/repeat unit; R.U.) = [IEC/(1000 × Fw (R.U.))]/[1 − (IEC/(1000 × Fw (SO_3_)))]

where Fw (R.U.) = 400.45, Fw (SO_3_) = 80.06.

The water uptake (W.U.) of the membrane was calculated using the following formula.

W.U. (%) = [(W_wet_ − W_dry_) × 100]/W_dry_

The mass of the dry membrane (W_dry_) was obtained by placing it in a drying oven at 80 °C for 24 h. The mass of the membrane containing water (W_wet_) was obtained by soaking it in boiling water for 1 h, immediately removing it, eliminating the surface water, and measuring the mass.

The degree of crosslinking of the membrane was calculated using the following method.

D_crosslink_ (%) = [(IEC_before annealing_ − IEC_after annealing_) × 100]/IEC_before annealing_

λ of the membrane was calculated using the following formula.

λ ([H_2_O]/[SO_3_H]) = [1000(W_wet_ − W_dry_)]/18W_dry_IEC = (10 × W.U.)/(18 × IEC)

A stress–strain test on the membrane was performed at room temperature using a tensile testing machine (Shimadzu Co., Ltd., EZ-S, Kyoto, Japan). The sample was cut using a Super Dumbbell Cutter SDMP-100 (manufactured by Dumbbell Co., Ltd., Saitama, Japan).

The conductivity of the CSPPSU membrane was determined by measuring the impedance using an MTS740 membrane test system (MTS, Scribner Associates, Inc., Southern Pines, NC, USA) and the four-probe method in a frequency range of 1 Hz to 1 MHz and a peak-to-peak voltage of 10 mV. The electrode used was a carbon paper electrode (electrode area = 0.9 cm^2^, Scribner Associates, Inc., Southern Pines, NC, USA) exclusively for the MTS740 device.

IrO_2_ (1 mg/cm^2^) and Pt/C (Pt, 1 mg/cm^2^) catalysts on PTFE sheets were purchased from Chemix Co. Ltd., Kanagawa, Japan. Nafion was used as the catalyst ionomer. The area of the electrode was 1 cm^2^.

The MEAs were made by using a decal method and hot pressing (Model A-010D, FC-R&D Company, Kanagawa, Japan) at 130 °C for the Nafion 115 membrane and 165 °C for the CSPPSU membrane at 9.8 kN for 10 min.

**Figure 1:**
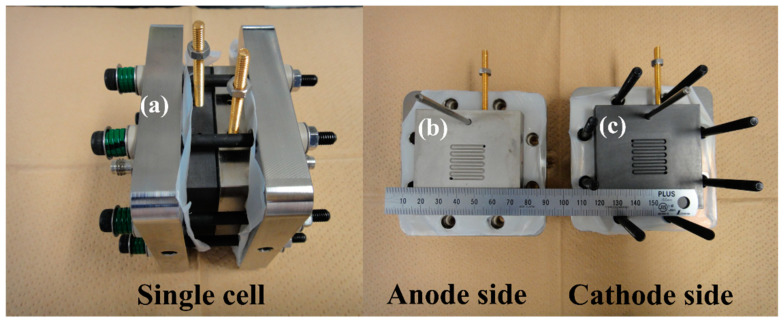
Photographs of a single cell, the anode side, and the cathode side; (**a**) SUS316L end plate, (**b**) Pt/Ti separator plate, and (**c**) carbon separator plate.

An 8.8 cm × 8.8 cm SUS316L end plate was used in the water electrolysis evaluation cell (Ulimeng Eng Co., Ltd., Chungbuk, Korea). In addition, the separator plate (6 cm × 6 cm) has a 2 cm × 2 cm serpentine channel. A Ti/Pt separator was used on the anode side, and a carbon separator was used on the cathode side. Photographs of a single cell, the anode side, and the cathode side are shown in Figure 1. No porous transport layer (PTL) was used on the anode side, and carbon cloth GDL (EIWA Corporation, Tokyo, Japan) was used on the cathode side. The water on the anode and cathode sides was heated (80 °C) using an oil bath and supplied to the cell at a rate of 2.0 mL/min via a pump. Water was supplied by circulation, and the outlet pressures on the anode and cathode sides were atmospheric pressure. The single cell was placed in a dry oven (DX301, Yamato Scientific CO., Ltd., Tokyo, Japan), and the oven temperature was used as the temperature of the single cell.

For water electrolysis, electrochemical measurements were performed at cell temperatures of 80, 100, 120, and 150 °C. For electrochemical measurements, current–voltage and EIS characteristics were investigated using a 1280C electrochemical test system (Solartron Analytical, Farnborough, UK) with a 20 A booster (Toyo corporation, Tokyo, Japan). The applied voltage was 1.4–2.0 V, and the current was measured while sweeping at 10 mV/s. The data were measured 2–3 times under these conditions, and the values with stable current-voltage characteristics were used. EIS was measured at 1.5 V in the frequency range of 1 Hz–20 kHz. Moreover, we evaluated the current characteristics over time at a cell temperature of 120 °C and 1.7 V.

## 3. Results

The properties of the Nafion 115 and CSPPSU membranes are summarized in Table 1. The IEC of the CSPPSU membrane is approximately 2, which is two times higher than that of Nafion 115. The WU of the CSPPSU membrane is equivalent to Nafion 115, but the λ value is half. The conductivity is about four times lower than Nafion 115. The tensile strength of the CSPPSU membrane is higher than Nafion 115, and the tensile elongation is lower than Nafion 115. The elastic modulus of the CSPPSU membrane is about five times larger than that of Nafion 115. The low λ and conductivities of the CSPPSU membranes were attributed to the rigidity of the CSPPSU polymer structure. We are investigating ways to improve the conductivity by introducing more sulfonic acid groups per unit of PPSU and plan to report on this in the future. The hydration stability of the CSPPSU membrane showed no decrease in IEC and conductivity even after 2184 h of autoclaving at 150 °C [19]. However, the hydration stability of the Nafion 115 membrane changed significantly after 2184 h of autoclaving at 150 °C. Specifically, the conductivity of the Nafion 115 membrane decreased by 41% at 80 °C and 90% RH.

**Figure 2:**
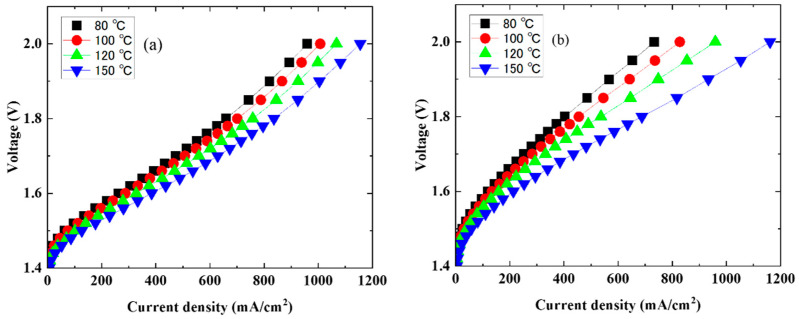
Polarization curves: (**a**) Nafion 115 and (**b**) CSPPSU membranes at different operation temperatures.

The water electrolysis properties of the Nafion 115 and CSPPSU membranes were investigated by fabricating MEAs using a decal method (Figure 2). Figure 2a,b show the current density-voltage (I–V) characteristics at cell temperatures of 80–150 °C using MEAs with the Nafion 115 and CSPPSU membranes, respectively. The current density increased, and the voltage decreased with an increase in the cell temperature. The maximum current density of the MEAs with the Nafion 115 and CSPPSU membranes was 1.2 A/cm^2^ at a cell temperature of 150 °C and a voltage of 2 V. The voltage and current density values at each temperature are summarized in Table 2. The same trends have been reported at lower electrolysis voltages, higher current density, and lower cell resistance due to higher operating temperatures [21,28,29]. From these results, the high-temperature operation of water electrolysis effectively reduces the overvoltage of the entire cell, and high current densities are obtained at low voltage.

**Figure 3:**
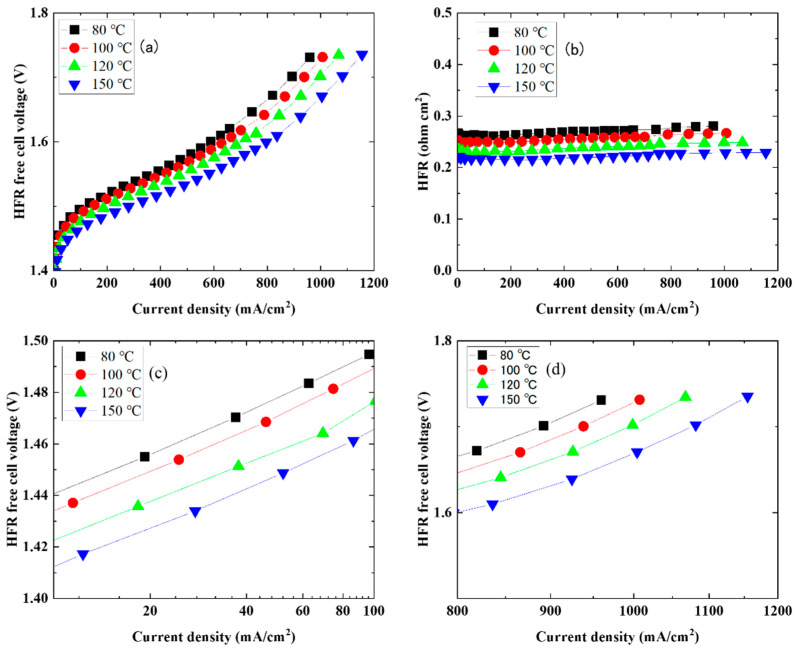
Electrochemical performance analysis of Nafion 115 membrane (Figure 2a): (**a**) Polarization curves of the HFR-free cell; (**b**) HFR vs. current density; (**c**) HFR-free polarization data at low current densities, plotted on a logarithmic scale and (**d**) at current densities between 800 and 1200 mA/cm^2^.

Figure 3 shows the results of analyzing the IV characteristics of the Nafion 115 membrane (Figure 2a). Figure 3a shows the I–V characteristics of the HFR-free membrane, Figure 3b shows the high-frequency-resistance (HFR) and current density characteristics, and Figure 3c,d show the current density and I–V characteristics of HFR free on a log scale. HFR decreased with an increase in the cell temperature (Figure 3b). In addition, due to the low current density on the log scale and the I–V characteristics of the HFR-free cell, the HFR-free voltage decreased as the cell temperature increased (Figure 3c). It is thought that the higher operating temperature improves the catalyst activity, lowers the reaction overvoltage, and reduces the HFR free voltage [22]. On the other hand, the HFR-free voltage on a log scale of 960 mA/cm^2^ decreased from 1.73 V at a cell temperature of 80 °C to 1.65 V at 150 °C. The decrease in the HFR-free voltage at a high current density due to an increase in operating temperature is thought to be due to a decrease in the mass transport loss [22].

**Figure 4:**
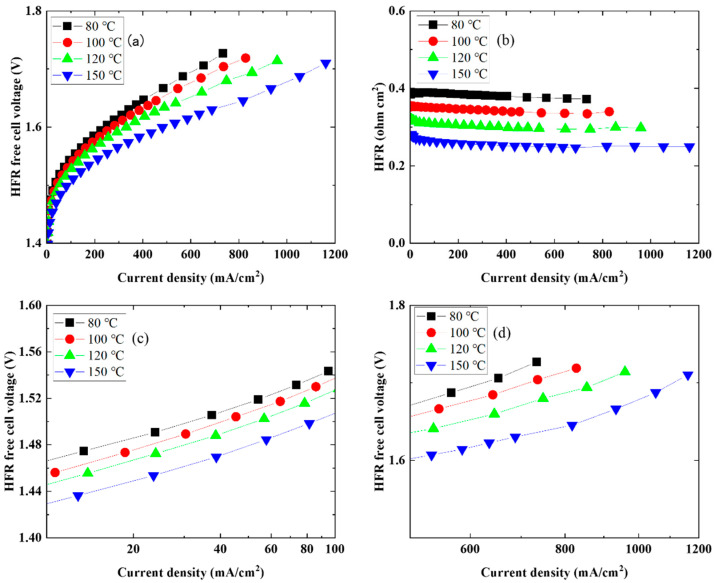
Electrochemical performance analysis of CSPPSU membrane (Figure 2b): (**a**) Polarization curves of HFR-free cell; (**b**) HFR over current density; (**c**) HFR-free polarization data at low current densities, plotted on a logarithmic scale and (**d**) at current densities between 500 and 1200 mA/cm^2^.

Figure 4 shows the results of analyzing the I–V characteristics of the CSPPSU membrane (Figure 2b). Figure 4a shows the I–V characteristics of the HFR-free cell, Figure 4b shows the HFR and current density characteristics, and Figure 4c and d show the current density and I–V characteristics of HFR free on a log scale. The characteristics of HFR free voltage and current density due to the increase in cell temperature of water electrolysis using the MEA with the CSPPSU membrane showed the same trend as when using the Nafion 115 membrane, as shown in Figure 3. Although the conductivity of the CSPPSU membrane was about four times lower than that of the Nafion 115 membrane, a similar current density was obtained from high-temperature water electrolysis at 150 °C (Table 1 and Table 2). Thus, the CSPPSU membrane with high glass transition temperature [27] can be used as an electrolyte membrane for high-temperature water electrolysis.

**Figure 5:**
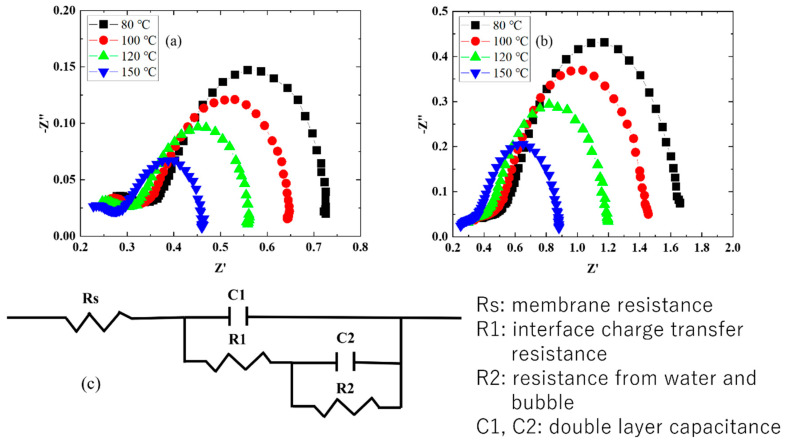
Nyquist plots measured at different operation temperatures of cells with (**a**) Nafion 115 and (**b**) CSPPSU membranes; (**c**) equivalent circuit used to fit the EIS data.

After I–V measurements at each temperature using a water electrolysis cell (Figure 2), EIS was performed. Figure 5a,b show Nyquist plots depending on the temperature after I–V measurements at 2 V on the cells containing the Nafion 115 and CSPPSU membranes. Figure 5c shows the model equivalent circuit used to analyze the Nyquist plot. In the Nyquist plot for the cells using Nafion 115 and CSPPSU membranes, the impedance decreased as the cell temperature increased. These results are the I–V characteristics under high-temperature operation, and they are consistent with the results when the voltage decreased and the current density increased as the cell temperature increased (Figure 2).

In other words, high-temperature operation lowered the cell overvoltage and membrane resistance, resulting in a high current density at low voltage. The results obtained by fitting the Nyquist plot using a model equivalent circuit are summarized in Table 3. It is difficult to fit using the membrane resistance (Rs), charge transfer resistance (Rct), double layer capacitance (Cdl), and constant phase element (Cpe) [18,19,20,23,30], which are commonly used in equivalent circuits. Therefore, fitting was performed using the equivalent circuit shown in Figure 5c. The resistance (Rs) of the cells with the Nafion 115 and CSPPSU membranes decreased as the cell temperature increased. In addition, R1 and R2 decreased as the cell temperature increased. A more detailed study is required regarding the components of R1 and R2. In this study, R1 was the interfacial charge transfer resistance, and R2 was the resistance due to water (water vapor) and bubbles (oxygen) generated at the membrane and electrode interface or between the IrO_2_ catalyst layer and the ionomer. Since the R2 and C2 components were not present when a porous IrO_2_ electrode catalyst was used [18,19], they are the EIS characteristics of an MEA cell fabricated using the decal method.

**Figure 6:**
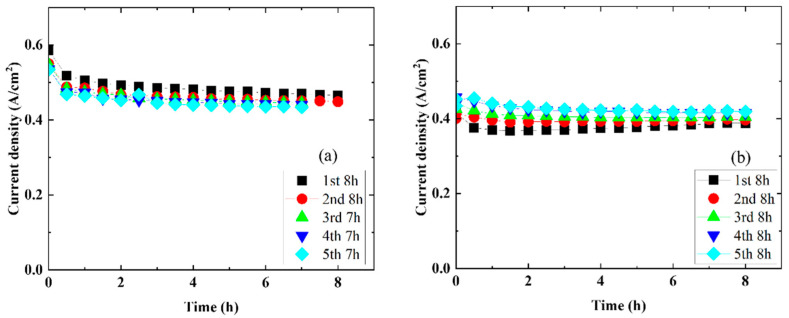
Time dependence of the single cell with (**a**) Nafion 115 and (**b**) CSPPSU membranes at 120 °C and 1.7 V.

Figure 6 shows the time dependence of the current density for water electrolysis conducted five times at a cell temperature of 120 °C and a voltage of 1.7 V. The temporal stability of the cell using the Nafion 115 membrane decreased by approximately 8.5% (0.47 A/cm^2^ in the first 7 h; 0.43 A/cm^2^ in the fifth 7 h) from the first to the fifth repeated measurement. On the other hand, regarding the temporal stability of the cell using the CSPPSU membrane, the current density did not decrease even after repeated measurements from the first to the fifth time. From the EIS properties before and after the time dependence of the Nafion 115 and CSPPSU membranes, the resistance of the Nafion 115 membrane is not significantly different before and after, but there is a difference in the resistance of the CSPPSU membrane, which may be due to the thinner CSPPSU membrane.

**Figure 7:**
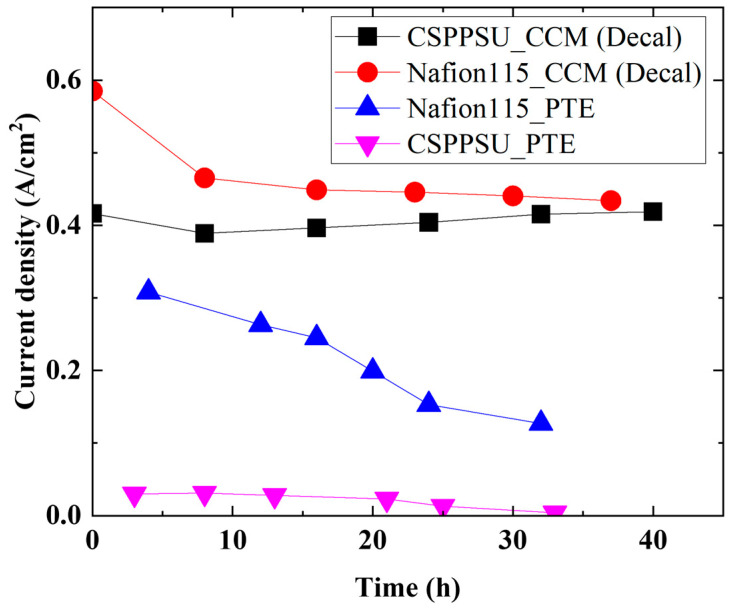
Comparison of the time dependence of a single cell of an MEA with a Nafion 115 or a CSPPSU membrane made using a decal method and a porous IrO_2_ electrode at 120 °C and 1.7 V [18,19].

Figure 7 shows a comparison of the time dependence of a single cell of an MEA with a Nafion 115 or a CSPPSU membrane made using the decal method and a porous IrO_2_ electrode (PTE) at 120 °C and 1.7 V. During the measurement of the water electrolysis properties of the MEA with an electrode (PTE) [18,19] made by coating IrO_2_ on the surface of porous Ti by electroplating, the current density decreased with time. In particular, the decrease in current density over time was much greater for the MEA with the CSPPSU membrane than with the Nafion 115 membrane. On the other hand, the stability of the cells with the CSPPSU and Nafion 115 membranes made using the decal method during water electrolysis was largely improved. Table 4 summarizes this research and literature evaluating water electrolysis stability at temperatures above 100 °C. The stability of the cell with the CSPPSU membrane made using the decal method during water electrolysis was found to be more durable than those reported in other literature, and it is expected to be used as a high-temperature electrolyte membrane.

## 4. Conclusions

The decal method was applied to obtain the time at which the CSPPSU membrane was stable in high-temperature water electrolysis. It was found that the stability of the CSPPSU and the Nafion 115 membranes was greatly improved by adopting the decal method compared to the PTE method used in the previous paper. And also, these results indicate that the CSPPSU membrane can be used as a high-temperature electrolyte membrane and that the decal method is effective for preparing MEAs. On the other hand, when the cell was disassembled after 40 h of stability evaluation, the CSPPSU membrane and the catalyst on the anode side were found to have peeled off. It became clear that an ionomer that is compatible with the CSPPSU membrane is necessary to ensure the long-term stability of water electrolysis using the CSPPSU membrane.

## Figures and Tables

**Table 1:** The Nafion 115 and CSPPSU membranes.

	Nafion 115	CSPPSU
IEC (meq/g), 25 °C	∼1.0	1.8
Crosslink rate (%), 25 °C	-	50
Water uptake (%), 100 °C	38	37
l, 25 °C	21.1	11.4
Elongation strength (MPa), 25 °C	31	53
Elongation stain (%), 25 °C	200	33
Flexural modulus (MPa), 25 °C	196	986
Conductivity, 80 °C, 90%RH, mS/cm	47	12

**Table 2:** Current–voltage properties at different operating temperatures using Nafion 115 and CSPPSU membranes.

Cell Temperature (°C)	Nafion 115		CSPPSU	
	mA/cm^2^ at 1.8 V	mA/cm^2^ at 2 V	mA/cm^2^ at 1.8 V	mA/cm^2^ at 2 V
80	659	960	403	734
100	701	1008	455	828
120	758	1068	537	959
150	836	1155	688	1161

**Table 3:** Parameters obtained from the Nyquist plot (Figure 4a,b) fitted to the equipment circuit shown in Figure 4c.

	Nafion 115				CSPPSU			
	Cell Temperature (°C)				Cell Temperature (°C)			
	80	100	120	150	80	100	120	150
Rs (ohm)	0.27	0.26	0.24	0.22	0.37	0.33	0.29	0.24
R1 (ohm)	0.1	0.08	0.07	0.06	0.19	0.16	0.14	0.12
C1 (mF)	0.474	0.481	0.417	0.412	0.447	0.354	0.378	0.422
R2 (ohm)	0.36	0.31	0.25	0.18	1.1	0.97	0.76	0.52
C2 (mF)	90.8	78.0	71.0	68.8	40.9	36.3	34.3	34.7

**Table 4:** Degradation experiments from the literature involving high temperature (>90 °C) operation.

Ref.	Temp. (°C)	Pressure (Bar)	Opera. Cond.	Operating Time (h)	Membrane	Catalyst (Loading)		PTL or PTE or CCM	GDL	Degradation Rate
						Anode (mg/cm^2^)	Cathode (mg/cm^2^)			
This study	120	1	1.7 V, 0.58 A/cm^2^	37	Nafion 115	IrO_2_ (1)	Pt (1)	CCM (Decal), Pt backing	Carbon cloth (0.291 mm)	−4.1 mA/cm^2^/h
This study	120	1	1.7 V, 0.42 A/cm^2^	40	CSPPSU	IrO_2_ (1)	Pt (1)	CCM (Decal), Pt backing	Carbon cloth (0.291 mm)	0 mA/cm^2^/h
[18]	120	1	1.7 V, 0.31 A/cm^2^	8	Nafion 115	IrO_2_ (7.5)	Pt (0.3)	PTE: Ti powder porous sheets (plating) (500 mm)	Carbon fiber paper (0.235 mm)	−18.9 mA/cm^2^/h
[19]	120	1	1.7 V, 0.03 A/cm^2^	8	CSPPSU	IrO_2_ (7.5)	Pt (0.3)	PTE: Ti powder porous sheets (plating) (500 mm)	Carbon fiber paper (0.235 mm)	Separation of membrane and electrode
[31]	120	3	1.5 V, 0.4 A/cm^2^	300	Composite SiO_2_-Nafion	IrO_2_ (5)	Pt (0.8)	CCM, Ti backing	Carbon cloth	−0.39 mA/cm^2^/h
[32]	130	1	1.9 V, 0.35 A/cm^2^	~1200	PA-doped Aquivion	IrO_2_ (0.7)	Pt (0.7)	CCM (Decal) Ta-coated stainless steel felt	Non-woven carbon cloth	+0.09 mV/h
[33]	150	5	1.7 V, 1.2 A/cm^2^	160	Nafion 117	IrO_2_ (0.8)	Pt (0.5)	PTE: Ti felt	Pt-based GDE	−2.5 mA/cm^2^/h
[34]	120	2.5	1.72 V, 1.25 A/cm^2^	300 (e-dep) or 150 (spray)	Nafion 212	IrO_2_ (0.4)	Pt (0.4)	PTE: Ti-based	Carbon fiber paper (0.325 mm)	−1.5 mA/cm^2^/h −3.93 mA/cm^2^/h

## Data Availability

The data presented in this study are contained within the article, further inquiries can be directed to the corresponding author.
